# The synergistic effect of salt-tolerant *Stutzerimonas stutzeri* and arbuscular mycorrhizal fungi alleviates salinity stress in soybean (*Glycine max*, L.)

**DOI:** 10.1007/s11274-026-04844-x

**Published:** 2026-03-11

**Authors:** Ahmed M. El-Sawah, Randa M. Zaki, Eman H. Ashour, Aida H. Afify

**Affiliations:** Department of Agricultural Microbiology, Faculty of Agriculture, Mansoura University, Mansoura, 35516 Egypt

**Keywords:** Arbuscular mycorrhizal fungi, Salinity, Salt-tolerant bacteria, Soybean

## Abstract

Using saltwater in agriculture has emerged as a solution that may help to address the issue of water supply for agriculture in light of current climate change. On the other side, salinity has a major impact on crop yield, particularly sensitive crops, and may have long-term implications for soil sustainability. Therefore, this study aimed to test the potential impact of a salt-tolerant bacterium, “*Stutzerimonas stutzeri* PV248835” along with arbuscular mycorrhizal fungi in alleviating salinity stress in soybean plants, which is regarded as one of the most important economic plants in Egypt. Our results showed that the arbuscular mycorrhizal species could still colonize soybean roots under both salinity levels (50 mM NaCl and 100 mM NaCl), and the combined application of (AMF + *S. stutzeri*) was more pronounced in enhancing the mycorrhizal indices (F%, frequency; M%, intensity; and A%, arbuscular development) in the roots. Moreover, the count of bacteria in the soybean rhizosphere was enhanced due to the bioinoculant applications under salinity stress. Furthermore, using bioinoculants improves soybeans’ morphological parameters, photosynthetic-related pigments, and nutrient uptake (N, P, and K) significantly at *p* ≤ 0.05 under salinity stress. While bio-inoculation leads to reduced Na uptake and Na^+^/K^+^ ratio in plant tissues compared to the control plants. In addition, bio-inoculation activates the antioxidant defense system in terms of PPO, POX, and CAT activities, while reduced proline accumulation was observed. The findings suggest that applying a combined treatment of (AMF + *S. stutzeri*) is an environmentally friendly way to alleviate salt stress in soybean cultivation while also preserving soil sustainability.

## Introduction

Egypt confronts a big problem in the form of water shortage for agriculture, which might have a significant influence on agricultural output and the ability to bridge the food gap caused by continuous population increase (Negm et al. 2019). Using saline water along with fresh water is one approach for addressing this deficit; however, irrigation with saline water has a negative impact on plant growth and productivity, as well as increasing soil salinity over time (AsadUllah et al. 2021; El-Sawah et al. 2023).

Salinity is considered a form of abiotic stress that has morphological, physiological, and biochemical effects on plants (Arif et al. 2020; Pandit et al. 2024). Salinity negatively impacts seed germination (Shu et al. 2017; Irik and Bikmaz 2024), plant growth (Sarioğlu 2025), photosynthetic machinery by decreasing photosynthetic pigments content such as chlorophyll and carotenoids (El-Sawah et al. 2023), transpiration, and gaseous exchange (Sperling et al. 2014). Soil salinization has a detrimental impact on plant water relations because it decreases soil water potential, which reduces leaf water potential, plant turgor, and causes osmotic stress (Soto Gonzáles et al. 2021). Salinization leads to nutritional imbalance in plant tissues. For instance, salinity stress leads to K^+^ loss in plant cells, while Na^+^ and Cl^−^ ions accumulate more quickly (Zhao et al. 2021). Additionally, salinization increases reactive oxygen species (ROS) in plants, causing oxidative stress, which affects normal cellular functioning by damaging DNA, protein, and lipids in plant tissues (Kesawat et al. 2023).

Salt-tolerant bacteria (STB), also known as halotolerant bacteria, are microorganisms that can thrive in environments with high salt concentrations, such as saline soils (Ma et al. 2022). These bacteria can alter the physiological and biochemical processes of inoculated plants under salinity stress through various mechanisms that directly or indirectly contribute to the alleviation of salt stress in plants, such as the production of phytohormones, exopolysaccharides, proline, antioxidants, and phosphate solubilization (Zaki et al. 2025). Besides, arbuscular mycorrhizal fungi (AMF) are species of fungi that form an intimate symbiotic relationship with plants in various soils (Nader et al. 2024; Peng et al. 2025). With the help of AMF colonization, the structure of roots could be improved, in particular under high levels of salinity (El-Sawah et al. 2023). AMF can significantly improve phosphorus acquisition by absorbing more P within their hyphae, producing phosphatase enzyme, which liberates P from its bound form and increases P availability in the soil, and maintaining intrinsic phosphate concentration (Pi) within the hyphae by forming polyphosphates (Evelin et al. 2019). In addition, AMF enhances K uptake, controlling Na^+^ translocation to aboveground parts, and regulating Na^+^ concentrations in plant tissues (Evelin et al. 2019; Boorboori and Lackóová 2025). This is due to the ability of mycorrhizal plants to sequester Na^+^ into vacuoles or exclude it from the cytosol. As well as AMF also help the host plant retract Na^+^ from the xylem and redirect it away from photosynthetic tissues and toward the roots (Evelin et al. 2019). Hence, using such beneficial microorganisms could be a cost-effective and easy-to-implement technique that could help farmers to mitigate salinity stress on economically important plants.

Soybean (*Glycine max* L.) is an economically important plant cultivated in Egypt for vegetable protein and oil (Liu et al. 2020; Grassini et al. 2021); however, salinity stress reduces the growth and productivity of this important crop (Kokebie et al. 2024). In this regard, we aimed to test the impact of salt-tolerant bacteria (STB) and/or AMF on the alleviation of salinity stress on soybean plants. We hypothesized that inoculating soybean plants with STB and/or AMF could modulate the morphological, biochemical, and physiological traits of soybean plants and help these plants to tolerate salinity. To test this hypothesis, several traits have been tested to gain a better understanding of how such inoculation with beneficial microorganisms could help soybean plants withstand salinity stress.

## Materials and methods

### Arbuscular mycorrhizal fungi (AMF)

The inoculum of AMF contained a mixture of different spores of *Glomus clarum*, *Funneliformis mosseae*, and *Gigaspora margarita*. The spores were previously trapped for 6 months using Sudan grass plant as a host (approximately 70% colonization level), then the colonized roots of Sudan grass and the trapped soil (approximately 50 spores per gram soil) were used as a mycorrhizal inoculum (El-Sawah et al. 2021).

### Salt-tolerant bacteria

*Stutzerimonas stutzeri* PV248835 was used as a salt-tolerant bacterium in this study. This strain was isolated from saline Egyptian soil in our previous study (Zaki et al. 2025). As well as this strain could produce indole acetic acid (IAA), gibberellins (GA), P-solubilization, exopolysaccharides (EPS), proline, and antioxidants under salinity stress conditions (Zaki et al. 2025).

### Experimental setup

The impact of inoculation with AMF and/or *S. stutzeri* on alleviating salinity stress on the soybean plant was tested in a pot experiment. Soybean seeds (Giza 111) were sterilized with 1% NaClO, then the seeds (10 seeds/pot) were planted in plastic pots (each pot was filled with 10 kg of soil). After that, soybean plants were thinned to three healthy plants per pot. The experimental soil had a clay loam texture, electrical conductivity (1.64 dSm^− 1^), pH (8.05), organic matter (0.73%), N (67.56 mg kg^− 1^), P (19.66 mg kg^− 1^), and K (343 mg kg^− 1^). The experiment included 36 pots that were arranged in a complete randomized block design (CRBD) with four experimental settings as follows: (1) Control; (2) AMF; (3) *S. stutzeri*; and (4) AMF + *S. stutzeri*. Irrigation was performed with three concentrations of saline water (CK, 50 mM NaCl, and 100 mM NaCl).

### Microbial inoculation

To produce the mycorrhizal treatment, each pot was inoculated with five grams of mycorrhizal colonized soil and 0.5 g of colonized Sudan grass roots. To enhance AMF colonization of soybean roots, the inoculum was applied about 5 cm below the soil surface. The non-AMF plants were inoculated with autoclaved soil and colonized root inoculum to provide the same nutrients to soybean plants.

To produce the bacterial treatment, soybean seeds were soaked for 30 min in a liquid culture of *S. stutzeri* PV248835 containing 16% Arabic gum as an adhesive agent. The bacterium was previously grown on nutrient broth medium for 48 h at 30 °C until the bacterial density reached 10^8^ cfu per ml. To ensure the bacterial inoculation, an extra 10 mL of the bacterial inoculum was inoculated to each plant after 2 weeks of sowing.

### Staining and estimation of mycorrhizal colonization in soybean roots

Soybean root segments (approximately 1 cm) were stained with trypan blue according to Phillips and Hayman (1970), then the mycorrhizal indices in soybean roots were determined according to Trouvelot et al. (1986) using the Mycocalc software (https://www2.dijon.inrae.fr/mychintec/Mycocalc-prg/download.html).

### Determination of bacterial count in soybean rhizosphere

Pour-plate method was employed to determine the total bacterial count (TBC), phosphate-solubilizing bacterial count (PSBC), and potassium-releasing bacterial count (KRBC) in soybean rhizosphere on nutrient agar medium, Pikovskaya’s medium, and Alexandrov’s medium, respectively, according to the study of Zaki et al. (2025).

### Plant analysis

Three soybean plants from each treatment were randomly selected after 60 days of sowing. Morphological traits (shoot length, root length, shoot dry weight, root dry weight, and leaves number) according to the study of Nader et al. (2024). Leaf chlorophylls a, b, and carotenoids were determined according to the method of Lichtenthaler and Wellburn (1983). Total nitrogen was determined using the Kjeldahl method according to Jackson (2005), phosphorus content was determined according to Snell and Snell (1961); however, potassium and sodium were determined using atomic absorption spectroscopy according to Chapman and Pratt (1962). Proline content was determined in the fresh leaf samples using the ninhydrin colorimetric method according to Bates et al. (1973). Antioxidant enzyme activities, polyphenol oxidase (PPO), peroxidase (POX), and catalase (CAT) were determined according to Malik and Singh (1980), Hammerschmidt et al. (1982), and Aebi (1983), respectively.

### Statistical analysis

The data represent means ± standard deviation (SD). The data were analyzed using one-way analysis of variance (ANOVA) with SPSS version 27.0 (SPSS Inc., Chicago, IL, USA). Duncan’s multiple range test (*p* ≤ 0.05) was used to compare treatment means. The interaction between bio-inoculation and salinity stress was investigated using two-way ANOVA with the CoStat program. The principal component analysis and the polar heatmap of correlation were performed using Originpro 2024.

## Results

### Mycorrhizal colonization in soybean roots under salinity stress

Mean data of the mycorrhizal indices (F%, frequency; M%, intensity; and A%, arbuscular development) in soybean roots are shown in Table 1. Data revealed that the mycorrhizal colonization in soybean roots was gradually decreased as the concentrations of salinity increased; however, AMF still have the potential to colonize the roots under the high salinity concentration (100 mM NaCl). The combined application of (AMF + *S. stutzeri*) was more pronounced in enhancing the mycorrhizal indices (F, M, and A%) when compared to the individual application of AMF under both normal and salinity stress conditions. Additionally, no mycorrhizal indices were observed in the non-mycorrhizal soybean plants. These results indicate the ability of AMF to form a symbiotic relationship with soybean roots under salinity stress conditions.

**Table 1:** Mycorrhizal colonization indices (%) in the roots of soybean plants grown under salinity stress

Treatments		F%	M%	A%
Control	CK	–	–	–
	AMF	95 ± 0.44b	66.78 ± 0.72b	48.45 ± 0.55b
	*S. stutzeri*	–	–	–
	Combination	98 ± 1.25a	75 ± 0.92a	54.55 ± 1.2a
50 mM NaCl	CK	–	–	–
	AMF	80 ± 1.23d	43.33 ± 1.04d	28.67 ± 0.58c
	*S. stutzeri*	–	–	–
	Combination	87 ± 0.93c	54 ± 1.42c	29.25 ± 0.72c
100 mM NaCl	CK	–	–	–
	AMF	70 ± 1.45f	35 ± 0.9f	14.55 ± 0.97e
	*S. stutzeri*	–	–	–
	Combination	74.01 ± 0.85e	38.5 ± 0.57e	17 ± 0.8d
**Salinity**		***	***	***
**Bio-inoculation**		***	***	***
**Salinity × Bio-inoculation**		***	***	***

Data represent means ± SD; different letters within the same column indicate significance *, **, *** denote significant differences between factors at *P* ≤ 0.05, 0.01, and 0.001, respectively. Ck, Check; AMF, Arbuscular mycorrhizal fungi; F%, the frequency of root colonization; M%, the intensity of cortical colonization; A%, arbuscular frequency in roots

### Bacterial count in soybean rhizosphere under salinity stress

Mean data of the bacterial count (TBC, total bacterial count; PSBC, phosphate-solubilizing bacterial count; KRBC, potassium-releasing bacterial count) in soybean rhizosphere were shown in Table 2. In general, using bioinoculants (AMF and/or *S. stutzeri*) resulted in higher bacterial count under both the control and salinity stress conditions. On the other side, salinity reduces the bacterial counts; yet bioinoculant applications enhanced bacterial counts under saline stress when compared to their equivalent control. It was observed that the combined treatment at different salinity concentrations (50 mM NaCl and 100 mM NaCl) resulted in the highest bacterial counts. In addition, it was observed that inoculation with *S. stutzeri* enhanced total bacterial count more than AMF, and a similar direction was noted for phosphate-solubilizing bacterial count and potassium-releasing bacterial count. These results underscore the vital role of bioinoculation in enriching the soybean rhizosphere with beneficial microbes under abiotic stress conditions such as salinity.

**Table 2:** Counts of bacterial count (Log cfu/g dry soil) in the rhizosphere of bio-inoculated soybean under salinity stress

Treatments		TCB	PSBC	KRBC
Control	CK	8.012 ± 0.073 fg	5.911 ± 0.073ef	5.320 ± 0.033 cd
	AMF	8.143 ± 0.025de	6.096 ± 0.068 cd	5.383 ± 0.008bc
	*S. stutzeri*	8.401 ± 0.073b	6.223 ± 0.036b	5.421 ± 0.017ab
	Combination	8.522 ± 0.039a	6.363 ± 0.047a	5.499 ± 0.007a
50 mM NaCl	CK	7.796 ± 0.044 h	5.769 ± 0.046 g	5.185 ± 0.102e
	AMF	8.062 ± 0.056ef	5.890 ± 0.030e-g	5.326 ± 0.025 cd
	*S. stutzeri*	8.189 ± 0.015 cd	6.005 ± 0.039de	5.390 ± 0.011bc
	Combination	8.242 ± 0.023c	6.160 ± 0.130bc	5.461 ± 0.019ab
100 mM NaCl	CK	7.371 ± 0.067j	5.515 ± 0.146 h	5.038 ± 0.040f
	AMF	7.595 ± 0.066i	5.823 ± 0.028 fg	5.129 ± 0.030e
	*S. stutzeri*	7.932 ± 0.059 g	5.950 ± 0.030ef	5.305 ± 0.074d
	Combination	8.086 ± 0.078ef	6.014 ± 0.0201de	5.425 ± 0.025ab
**Salinity**		***	***	***
**Bio-inoculation**		***	***	***
**Salinity × Bio-inoculation**		***	ns	**

Data represent means ± SD; different letters within the same column indicate significance; ns, not significant; and *, **, *** denote significant differences between factors at *P* ≤ 0.05, 0.01, and 0.001, respectively. Ck, check; AMF, arbuscular mycorrhizal fungi; TBC, total bacterial count; PSBC, phosphate-solubilizing bacterial count; KRBC, potassium-releasing bacterial count

### Morphological traits of soybean plants under salinity stress

The morphological traits of the bioinoculated soybean plants grown under salinity stress are presented in Table 3. As expected, salt stress reduced plant growth and biomass accumulation compared to control soybean plants. Where the values of shoot length, root length, SDW, RDW, and leaves number under control, 50mM NaCl, and 100mM NaCl were recorded (20.83, 19.10, and 18.23 cm), (5.37, 3.80, and 1.50 cm), (0.28, 0.22, and 0.18 g/plant), (0.037, 0.024, and 0.020 g/plant), and (11.00, 8.67, and 7.00 leaves/plant), respectively. Microbial inoculation (combination > *S. stutzeri* > AMF) improves these traits at both salinity stress concentrations of 50mM NaCl and 100mM NaCl. Generally, the combined treatment gave the highest values of plant growth and biomass. The highest values of shoot length, root length, SDW, RDW, and leaves number under control, 50mM NaCl, and 100mM NaCl were recorded (27.70, 26.50, and 24.83 cm), (11.13, 9.57, and 6.17 cm), (0.60, 0.53, and 0.44 g/plant), (0.169, 0.089, and 0.057 g/plant), and (21.33, 18.00, and 14.00 leaves/plant), respectively. Such findings highlighted the importance of microbial bioinoculants, particularly the synergistic effect of (*S. stutzeri* and AMF) in improving soybean plant morphology.

**Table 3:** Morphological response of soybean plants grown under salinity stress

Treatments		Shoot Length (cm)	Root Length (cm)	SDW (g/plant)	RDW (g/plant)	No. of leaves/plant
Control	CK	20.83 ± 0.29f	5.37 ± 0.55de	0.28 ± 0.04 fg	0.037 ± 0.008d	11.00 ± 1.00e-g
	AMF	23.40 ± 1.82 cd	7.33 ± 0.76c	0.38 ± 0.08 cd	0.049 ± 0.002 cd	13.67 ± 2.08de
	*S. stutzeri*	23.63 ± 0.12bc	9.00 ± 0.50b	0.53 ± 0.03b	0.083 ± 0.030bc	16.67 ± 1.15bc
	Combination	27.70 ± 0.52a	11.13 ± 0.81a	0.60 ± 0.02a	0.169 ± 0.057a	21.33 ± 3.06a
50mM NaCl	CK	19.10 ± 0.53gh	3.80 ± 1.85f	0.22 ± 0.01gh	0.024 ± 0.001d	8.67 ± 1.15gh
	AMF	21.60 ± 0.10ef	5.83 ± 0.29c-e	0.31 ± 0.02ef	0.034 ± 0.003d	13.00 ± 1.00d-f
	*S. stutzeri*	23.50 ± 0.87 cd	6.93 ± 0.85 cd	0.37 ± 0.04de	0.046 ± 0.002 cd	15.00 ± 1.00 cd
	Combination	26.50 ± 0.50a	9.57 ± 1.10b	0.53 ± 0.04b	0.089 ± 0.021b	18.00 ± 0.00b
100mM NaCl	CK	18.23 ± 0.21 h	1.50 ± 0.50 g	0.18 ± 0.01 h	0.020 ± 0.002d	7.00 ± 1.73 h
	AMF	20.33 ± 1.04 fg	4.53 ± 0.50ef	0.27 ± 0.01 fg	0.028 ± 0.001d	10.33 ± 1.15 fg
	*S. stutzeri*	22.23 ± 0.49de	5.43 ± 0.51de	0.39 ± 0.05 cd	0.039 ± 0.002d	11.00 ± 0.00e-g
	Combination	24.83 ± 0.42b	6.17 ± 1.15c-e	0.44 ± 0.03c	0.057 ± 0.005b-d	14.00 ± 2.00 cd
**Salinity**		***	***	***	***	***
**Bio-inoculation**		***	***	***	***	***
**Salinity × Bio-inoculation**		ns	ns	ns	**	ns

Data represent means ± SD; different letters within the same column indicate significance; ns, not significant; and *, **, *** denote significant differences between factors at *p* ≤ 0.05, 0.01, and 0.001, respectively. Ck, check; AMF, arbuscular mycorrhizal fungi; SDW, shoot dry weight; RDW, root dry weight

### Photosynthetic-related pigments of soybean plants under salinity stress

**Fig. 1:**
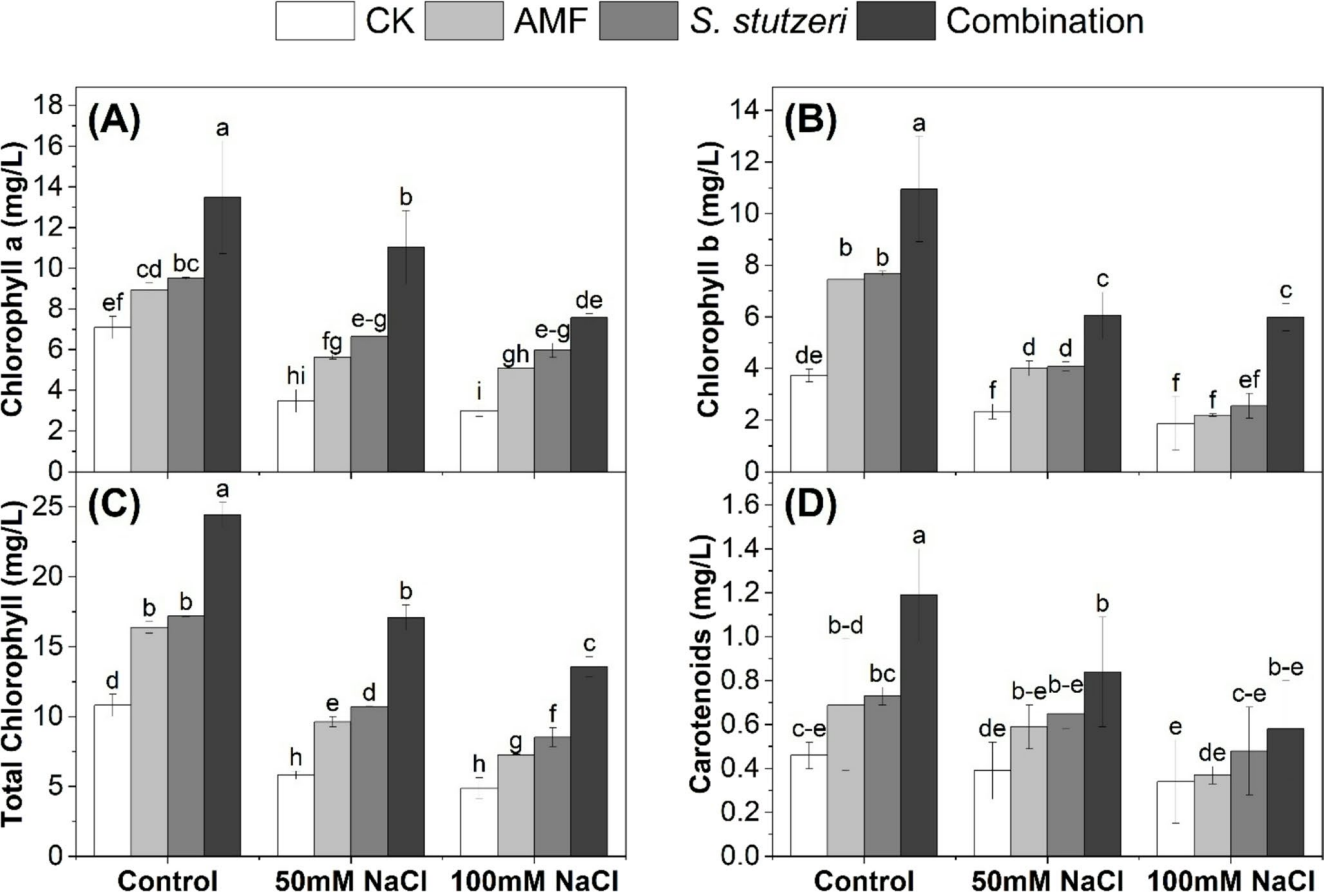
Changes in photosynthetic-related pigments in the leaves of soybean plants under salinity stress; (**A**): chlorophyll a, (**B**): chlorophyll b (**C**): total chlorophyll, (**D**): carotenoids. Data are means ± standard deviation; different letters indicate significant differences between means at *p* ≤ 0.05

Chlorophyll and carotenoid contents in the leaves of bioinoculated soybean plants grown under salinity stress are presented in Fig. 1. Salinity reduced the photosynthetic-related pigments in the leaves of soybean plants. Where the values of chlorophyll a, chlorophyll b, total chlorophyll, and carotenoids under control, 50mM NaCl, and 100mM NaCl were recorded (7.09, 3.48, and 3.00 mg/L), (3.73, 2.33, and 1.87 mg/L), (10.81, 5.81, and 4.87 mg/L), and (0.46, 0.39, and 0.34 mg/L), respectively. Microbial inoculation (combination > *S. stutzeri* > AMF) improves photosynthetic-related pigments at both salinity stress concentrations of 50mM NaCl and 100mM NaCl. The combined treatment gave the highest values of chlorophyll a, chlorophyll b, total chlorophyll and carotenoids under control, 50mM NaCl, and 100mM NaCl were recorded (13.48, 11.03, and 7.58 mg/L), (10.95, 6.06, and 5.99 mg/L), (24.43, 17.09, and 13.57 mg/L), and (1.19, 0.84, and 0.58 mg/L), respectively. Such findings underlined the potential of microbial bioinoculants, particularly the combination of *S. stutzeri* and AMF, to increase photosynthetic pigments in soybean plant leaves under salinity stress.

### Nutrient contents in soybean plants under salinity stress

**Fig. 2:**
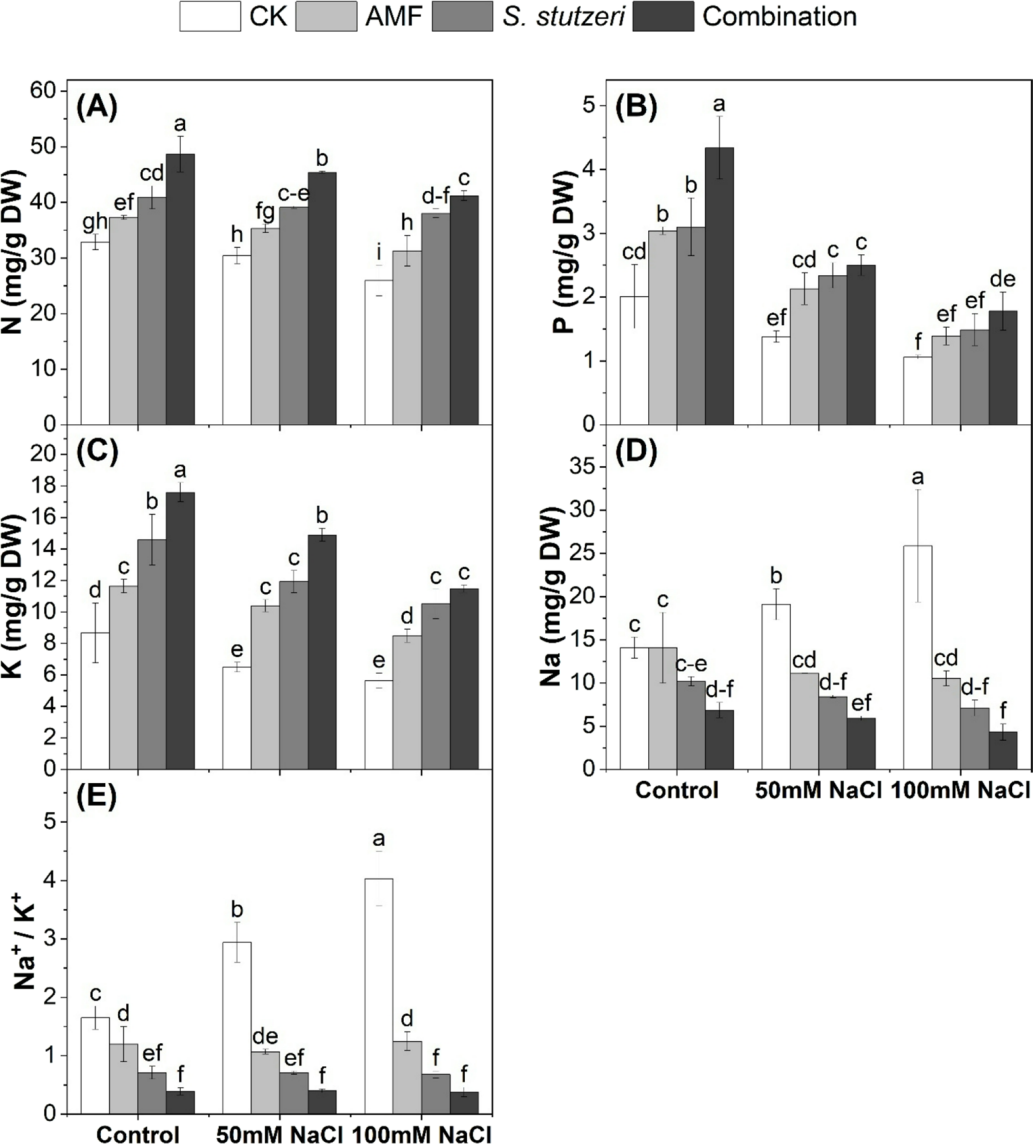
N, P, K, Na contents (mg/g DW) and Na^+^/K^+^ ratio in the shoots of soybean plants under salinity stress. (**A**): nitrogen, (**B**): phosphorus, (**C**): potassium, (**D**): sodium, (**E**), Na^+^/K^+^. Data represent means ± SD; different letters indicate significant differences between means at *P* ≤ 0.05. Ck, Check; AMF, Arbuscular mycorrhizal fungi

Nutrients (nitrogen, phosphorus, potassium, and sodium) and the sodium/potassium ratio in the shoots of bioinoculated soybean plants grown under salinity stress are presented in Fig. 2. Salinity reduced the N, P, and K contents in the shoots of soybean plants, and this decrease was more pronounced at 100 mM NaCl concentration. The contents of N, P, and K under control, 50mM NaCl, and 100mM NaCl were recorded (32.87, 30.43, and 25.96 mg/g DW), (2.01, 1.38, and 1.06 mg/g DW), and (8.67, 6.50, and 5.63 mg/g DW), respectively. Microbial inoculation (combination > *S. stutzeri* > AMF) increased N, P, and K at both salinity stress concentrations of 50mM NaCl and 100mM NaCl. The combined treatment gave the highest values of N, P, and K under control, 50mM NaCl, and 100mM NaCl were recorded (48.66, 45.40, and 41.22 mg/g DW), (4.34, 2.50, and 1.78 mg/g DW), and (17.60, 14.89, and 11.48 mg/g DW), respectively. On the other side, an increase was observed in Na content and the Na^+^/K^+^ ratio due to salinity stress Fig. 2. However, inoculation with combination > *S. stutzeri* > AMF could decrease Na uptake under control, 50mM NaCl, and 100mM NaCl. The combined treatment gave the lowest values of Na, which were recorded (6.85, 5.92, and 4.31 mg/g DW) under control, 50mM NaCl, and 100mM NaCl, respectively. In addition, the Na^+^/K^+^ ratio was decreased in the shoots of soybean plants due to microbial inoculation, and this was more pronounced in the combined treatment. These findings suggest that the combination of *S. stutzeri* and AMF could improve nutrient uptake while reducing Na uptake in soybean plants under salt stress.

### Proline content and antioxidant enzyme activities in soybean plants under salinity stress

**Fig. 3:**
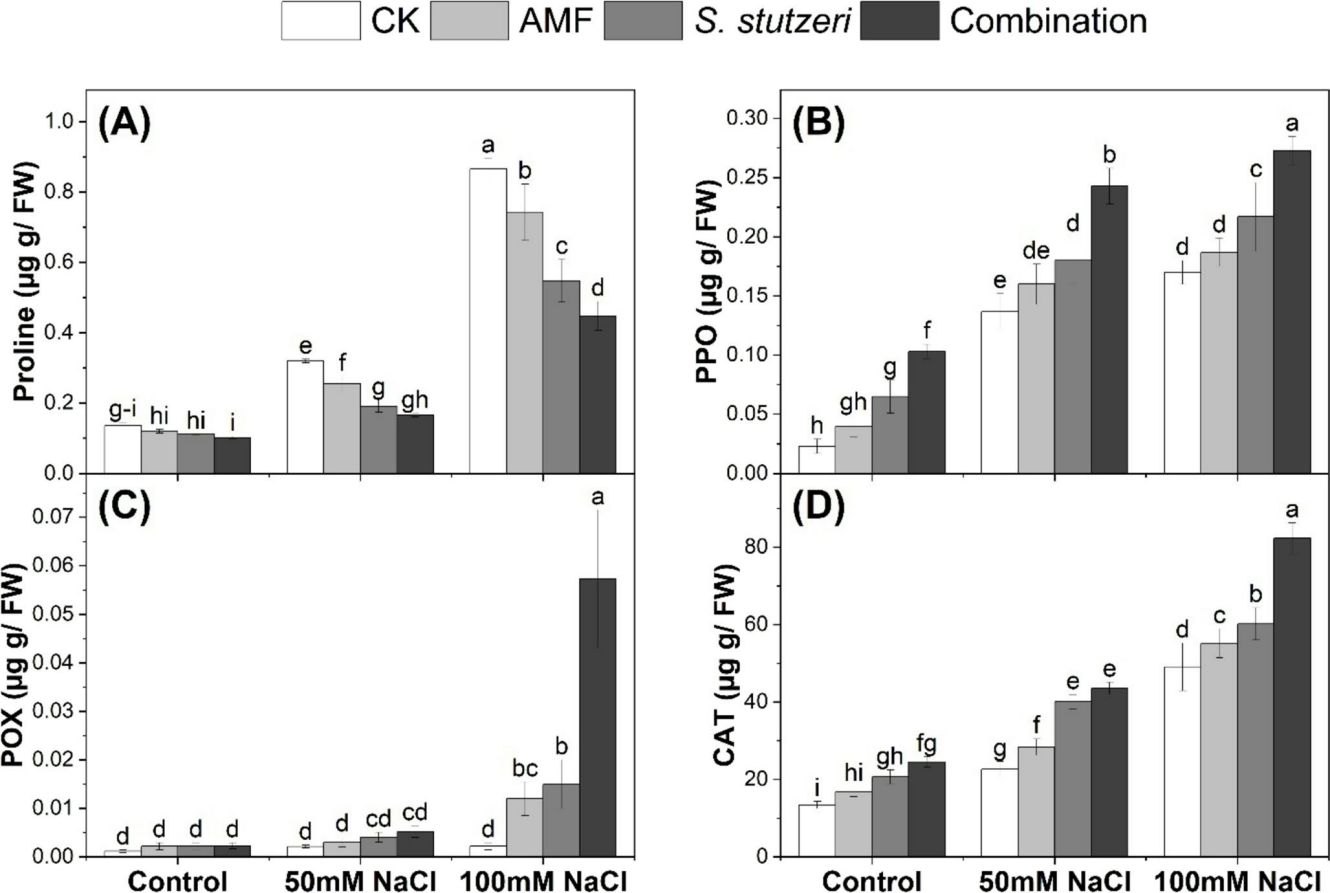
Proline content (µg g/FW) and antioxidants activity (U g/FW) in the leaves of soybean plants grown under either control or salinity stress conditions. (**A**): proline, (**B**): PPO, (**C**): POX, (**D**): CAT. Data represent means ± SD; different letters indicate significant differences between means at *P* ≤ 0.05. Ck, Check; AMF, Arbuscular mycorrhizal fungi

Proline content in the leaves of bioinoculated soybean plants grown under salinity stress is presented in Fig. 3A. Salinity significantly increased the contents of proline in the leaves of soybean plants, and this increase was more pronounced at 100 mM NaCl concentration. The contents of proline under control, 50mM NaCl, and 100mM NaCl were recorded (0.136, 0.320, and 0.866 mg/g FW), respectively. Microbial inoculation (combination > *S. stutzeri* > AMF) decreased proline content at both salinity stress concentrations of 50mM NaCl and 100mM NaCl. The combined treatment gave the lowest values of proline under control, 50mM NaCl, and 100mM NaCl were recorded (0.102, 0.166, and 0.448 mg/g FW), respectively.

The activity of antioxidant enzymes in the leaves of bioinoculated soybean plants grown under salinity stress is presented in Fig. 3B-D. The PPO and CAT activities have increased in response to salinity stress, but a slight increase was observed in POX. Microbial inoculation leads to an increase in the PPO, POX, and CAT, especially under 100mM NaCl. The maximum PPO, POX, and CAT activities were observed in the combined application under 100 mM NaCl.

The results indicate that microbial inoculation could reduce proline content and activate the antioxidant enzyme defense system to alleviate salinity stress in soybean plants.

### Evaluation of the impact of bioinoculants using principal component analysis and polar heatmap with dendrogram under salinity stress

**Fig. 4:**
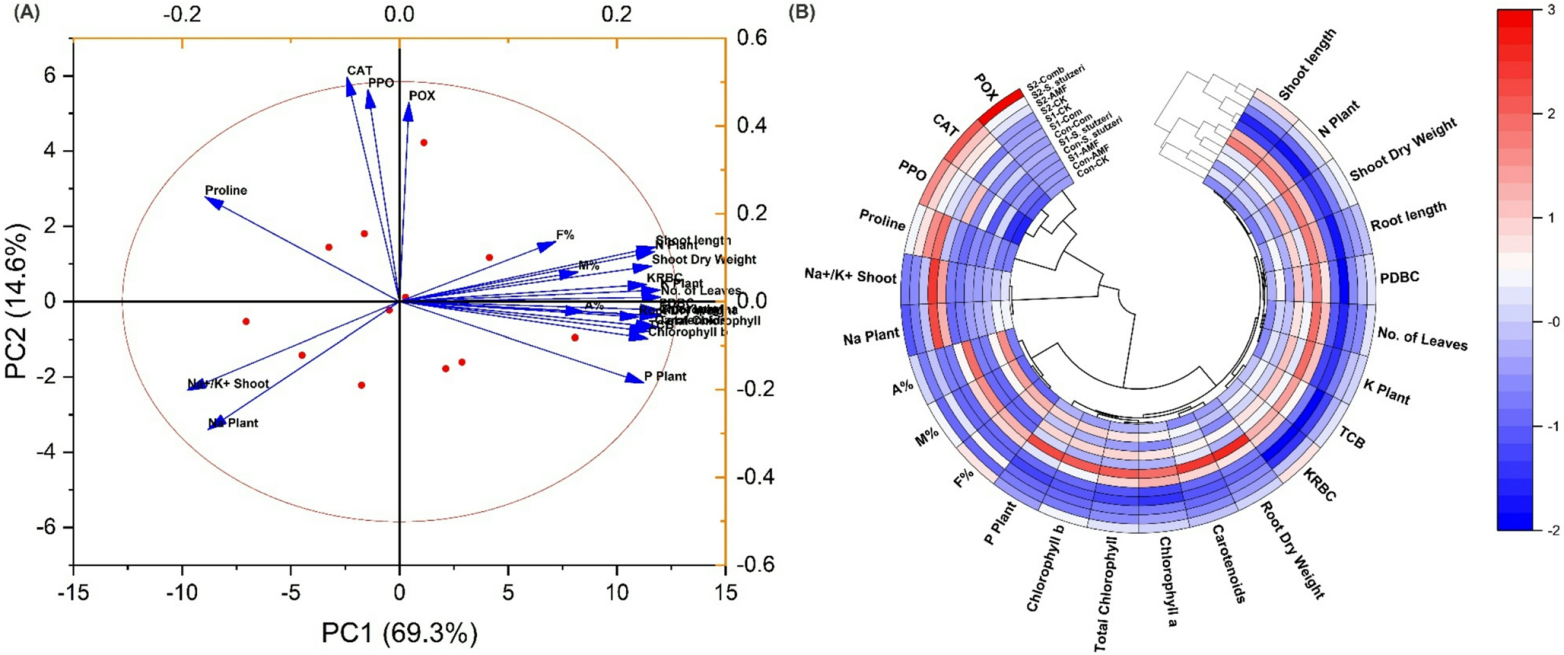
Changes in soybean growth related traits in bioinoculated soybean plants under salinity stress (**A**) Principal compoent analysis (PCA) (**B**) Polar Heatmap with dandrogam

The growth-related traits of bio-inoculated soybean plants under salinity stress were shown using PCA and polar heatmap with dendrogram (Fig. 4A&B). The PCA of the examined variables revealed two components (PC1 and PC2), accounting for 69.30% and 14.60% of the data variability, respectively. Additionally, the polar heatmap with dendrogram showed differences in the values of the examined variables of soybean-related traits. Such results highlight the significant impact of the used bioinoculants on alleviating salinity stress on soybean plants.

## Discussion

### Mycorrhizal colonization was enhanced by bio-inoculation under salinity stress

The indices of mycorrhizal colonization (F%, M%, and A%) decreased dramatically as salinity increased. Salinity stress could have hampered fungal growth, development, and structure, resulting in this decline. Similarly, several studies observed such a decline in mycorrhizal indices as affected by salinity stress (Juniper and Abbott 2006; El-Sawah et al. 2023; Zaki et al. 2025). Despite this reduction, the used species of AMF still have the ability to colonize soybean roots under the two levels (50mM NaCl and 100mM NaCl) of salinity stress, and this was more pronounced in the combined treatment, which gave the highest values of mycorrhizal indices under control, 50mM NaCl, and 100mM NaCl (Table 1). These results strengthen the positive impact of the species of AMF used in salinity alleviation through the symbiotic relationship that allows modification of soybean root architecture, and absorption of more water and nutrients by stressed soybean plants.

### Bacterial counts were enhanced by bio-inoculation under salinity stress

Several studies reported that salinity reduces microbial biomass, activity, and changes the community structure in the soil (Yan et al. 2015). Similarly, in this study, the bacterial counts (TCB, PSBC, and KRBC) decreased dramatically as salinity increased. This decrease could be attributed to the osmotic stress due to drying and lysis of cells (Yan et al. 2015). Microbial inoculation resulted in an improvement in bacterial counts under the two levels (50mM NaCl and 100mM NaCl) of salinity stress (Table 2). This increase could be attributed to the fact that microbial inoculation promotes the decomposition of organic matter in soil, which releases heat that promotes bacterial growth, as well as the availability of organic matter and nutrients that serve as a source of energy, thereby encouraging bacterial reproduction (Gao et al. 2020). Similar observations were reported by Zaki et al. (2025), who found an increase in the bacterial counts in maize rhizosphere after inoculation with bioinoculants under salinity stress. Such results indicate that the bioinoculants could contribute to the preservation of biological sustainability, which helps the availability of nutrients in the soil during salt stress.

### The morphological traits of soybean plants were improved by bio-inoculation under salinity stress

Soybeans’ morphological parameters, such as shoot length, root length, dry weight, and number of leaves, were reduced in response to salinity stress; however, bioinoculants’ application resulted in an improvement in these parameters under both unstressed and stressed plants (Table 3). Such improvement could be attributed to mycorrhizal symbiosis (Table 2), which increases water availability and nutrients concentration using hyphae under saline stress conditions. Moreover, the salt-tolerant bacterial strain (*S. stutzeri*) could produce plant growth-promoting substances such as IAA, GA, P-solubilized, proline, ESP, and antioxidant substances (Zaki et al. 2025). In addition, the combined application was found to be more pronounced when compared to the individual ones. This could be attributed to the synergistic effect between bacteria and mycorrhizal fungi. Our results are in line with several studies that reported the beneficial impact of PGPR and/or AMF on the morphological characteristics of soybean plants under salinity stress (Hashem et al. 2019; Abulfaraj and Jalal 2021; Khan et al. 2021; Kang et al. 2023; Lin et al. 2025; Pu et al. 2025).

### The photosynthetic-related pigments were improved in soybean plants under salinity stress

Our results showed that bio-inoculation with (AMF, *S. stutzeri*, and combination) could improve photosynthetic-related pigments content in soybean under salinity stress (Fig. 1). This could be attributed to nutrient availability, in particular P, which is very important for chlorophyll synthesis. AMF and *S. stutzeri* could convert insoluble phosphorus into forms that plants can absorb, which could increase P uptake in soybean plants. Thus, applying bioinoculants may be a potential technique for improving the photosynthetic apparatus under salinity stress. In this line, Hashem et al. (2019) found an improvement of chlorophyll a, b, and carotenoid content in soybean plants following AMF inoculation under salinity stress. A recent study by Lin et al. (2025) observed an increase in photosynthetic rate in soybean plants following inoculation with PGPR under salinity stress. However, our findings suggest that dual inoculation with AMF and *S. stutzeri* could be more effective in enhancing photosynthetic pigment content and, as a result, soybean growth under salinity stress.

### Bio-inoculation enhances nutrient contents and reduces Na^+^/K^+^ ratio in soybean plants under salinity stress

Our results showed that bio inoculation with (AMF, *S. stutzeri*, and combination) could enhance N, P, and K uptake as well as decrease Na^+^ and the Na^+^/K^+^ ratio under salinity stress (Fig. 2). This influence could be attributed to the ability of mycorrhiza and PGPR to provide nutrients such as phosphorus to the host plant (Gao et al. 2020). Moreover, inoculation with mycorrhiza and PGPR could enhance soybean nodulation, which in turn increases nitrogen content in soybean plants (Nader et al. 2024). In addition, AMF can restrict Na transport from roots to shoots in plants, decreasing Na^+^/K^+^ in shoots and compartmentalization of Na^+^ ions (Chandrasekaran et al. 2021; Wang et al. 2021). Similar observations were observed by Lin et al. (2025), who found PGPR minimized the accumulation of Na in the roots and leaves of the soybean plant; however, an increase in K was observed. Moreover, Pu et al. (2025) found that AMF inoculation increased P and K levels in soybean tissues while decreasing Na levels and raising K^+^/Na^+^ ratios under salt stress. These results strengthen the potential impact of bio-inoculants to support nutrients uptake and to reduce Na uptake in soybean plants under salinity stress.

### Bio-inoculation reduces proline content and activates the antioxidant defense system in soybean plants under salinity stress

Proline can act as a stress signal; therefore, plants may acquire more proline owing to stress or less proline as a result of decreasing stress, and this pattern may depend on several factors such as plant species, plant organs, and salinity level (Nader et al. 2024). Our results showed a decrease in proline accumulation due to bioinoculants, and this decrease was more pronounced in the combined application (Fig. 3A). Accumulation of low proline indicated that the treated plants were less exposed to salinity and did not require excessive proline accumulation to cope with salinity stress. Similarly, Echeverria et al. (2013) show a decreased proline accumulation in lotus plants following AMF significantly under salinity stress. Also, similar observations in soybean plants following PGPR applications under salinity stress (Abulfaraj and Jalal 2021). On the other hand, we observed a significant increase in the activities of antioxidant enzymes (PPO, POX, and CAT) due to bioinoculants application (Fig. 3B–D). Elevated enzyme activity indicates that soybean plants engage their defensive systems of antioxidant enzymes to scavenge those producing ROS during salt stress. These results are consistent with those obtained by Abulfaraj and Jalal (2021); Khan et al. (2021); Kang et al. (2023); Lin et al. (2025); Pu et al. (2025). Our results indicate that the elevated enzyme activities resulting from bioinoculants application can protect soybean plants from membrane damage caused by salinity stress by decreasing ROS.

## Conclusions

This advanced study intends to use bio-inoculants to reduce the harmful effects of irrigation water salinity on soybean plant growth. This study is critical for farmers because of the paucity of available irrigation water in the face of global climate change. This study proposes an environmentally friendly and simple technique for reducing salinity in soybean plants that employs a combined treatment of salt-tolerant bacteria and arbuscular mycorrhizal fungi. This treatment improved the morphological characteristics of soybean plants under salinity, enhanced the content of photosynthetic pigments, and increased the absorption of elements N, P, and K. It also reduced Na^+^ absorption and the Na^+^/K^+^ ratio in plant cells, activated the plant’s antioxidant defense system, and decreased proline accumulation in plant cells. Microbial treatments also resulted in significantly higher bacterial numbers in the rhizosphere compared to the control, hence increasing soil biological sustainability. Future research should focus on large field trials of soybean crops in various salinity-affected areas using a bio-inoculation system.

## Data Availability

The datasets used and analyzed in the current study are available from the corresponding author on reasonable request.
